# The efficacy of sleep lifestyle interventions for the management of overweight or obesity in children: a systematic review and meta-analysis

**DOI:** 10.1186/s12889-024-17708-6

**Published:** 2024-01-29

**Authors:** Ruyu Liu, Roger Figueroa, Heidi Vanden Brink, Colby J. Vorland, Sameera Auckburally, Lynn Johnson, Jessica Garay, Tamara Brown, Stacey Simon, Louisa Ells

**Affiliations:** 1 Division of Nutritional Sciences, College of Human Ecology, Cornell University, Ithaca, NY USA; 2 Department of Nutrition, Texas A&M University, College Station, TX USA; 3 Department of Epidemiology and Biostatistics, Indiana University, Bloomington, IN USA; 4 Department of Pediatric Endocrinology, Royal Manchester Children’s Hospital, Manchester, UK; 5 Cornell Statistical Consulting Unit, Cornell University, Ithaca, NY USA; 6 Falk College of Sport & Human Dynamics, Syracuse University, Syracuse, NY USA; 7 Obesity Institute, School of Health, Leeds Beckett University, Leeds, UK; 8 Pediatrics – Pulmonary Medicine, Children’s Hospital Colorado Anschutz Campus, Aurora, CO USA; 9 School of Clinical and Applied Sciences, Leeds Beckett University, Leeds, UK

**Keywords:** Body mass index, Nutrition, Diet, Childhood obesity, Body composition

## Abstract

**Background:**

Childhood obesity remains a significant public health concern. Sleep duration and quality among children and youth are suboptimal worldwide. Accumulating evidence suggests an association between inadequate sleep and obesity risk, yet it is unclear whether this relationship is causal. This systematic review examines the efficacy of sleep interventions alone or as a part of lifestyle interventions for the management of overweight or obesity among children and adolescents.

**Methods:**

A keyword/reference search was performed twice, in January 2021 and May 2022 in MEDLINE/PubMed, EMBASE/Ovid, PsycINFO/EBSCO, The Cochrane Library, Web of Science Core Collection/Web of Science, SciELO/Web of Science, and CINAHL/EBSCO. Study eligibility criteria included youth with overweight or obesity between 5 and 17, were RCTs or quasi-randomized, and focused on the treatment of overweight and obesity with a sleep behavior intervention component. Risk of bias was assessed using the Cochrane Risk of Bias assessment tool (RoB2). A Meta-analysis was conducted to estimate the effect of interventions with a sleep component on BMI. The study protocol was registered in PROSPERO (CRD42021233329).

**Results:**

A total of 8 studies (2 quasi-experiments, 6 RCTs) met inclusion criteria and accounted for 2,231 participants across 7 countries. Only one study design isolated the effect of sleep in the intervention and reported statistically significant decreases in weight and waist circumference compared to control, though we rated it at high risk of bias. Our meta-analysis showed no significant overall effect on children’s BMI as a result of participation in an intervention with a sleep component (Cohen’s d = 0.18, 95% CI= -0.04, 0.40, Z = 1.56, *P* = .11), though caution is warranted due to substantial heterogeneity observed across studies (Tau^2^ = 0.08; X^2^ = 23.05, df = 7; I^2^ = 83.73%).

**Conclusions:**

There were mixed results on the effect of sleep interventions across included studies on BMI, other weight-related outcomes, diet, physical activity, and sleep. Except for one study at low risk of bias, three were rated as ‘some concerns’ and four ‘high risk of bias’. Findings from this study highlight the need for additional RCTs isolating sleep as a component, focusing on children and adolescents living with overweight and obesity.

**Supplementary Information:**

The online version contains supplementary material available at 10.1186/s12889-024-17708-6.

## Background

Childhood obesity remains a significant public health concern worldwide. The prevalence of overweight and obesity in children ranges from 15.3 to 25.6% in Europe [1]. In the United States, the prevalence of obesity among children and youth aged 2 to 19 years has increased from 17.7 to 21.5% from 2011 to 2020 ^2^. Youth living with obesity are more likely to experience impairments in endocrine, metabolic, cardiovascular, pulmonary, neurological, immunologic, and gastrointestinal functions [2]. Therefore, there is an urgent need for innovative interventions that can mitigate the trajectory of accelerated weight gain among children.

Emerging evidence has demonstrated that short sleep duration and poor sleep quality are associated with childhood obesity [3–7]. Reduced sleep duration is associated with lower circulating leptin and increased ghrelin concentrations, which are anorexigenic and orexigenic hormones, respectively [8]. Thus, it is plausible that disrupted hormonal control of satiety via reduced sleep time results in increased food intake and induces hedonic eating rather than hunger-driven eating [9, 10]. Reduced sleep duration results in fatigue which is associated with reduced energy expenditure thereby contributing to a positive energy balance [3, 11–13]I. Obesity is also an independent risk factor for obstructive sleep apnea [14], potentially perpetuating a cycle of disrupted sleep and weight gain in adolescents. Thus, there is significant observational and mechanistic evidence that reduced sleep duration and obesity are related and that interventions targeting sleep duration may be efficacious for the treatment of overweight and obesity in children and adolescents. A randomized cross-over study elucidating the relationship between sleep duration and dietary intake corroborates this notion; children in the increased sleep phase of the study exhibited reduced caloric intake and reduced weight [15]. In addition to sleep duration, sleep quality is also emerging as a modifiable lifestyle factor associated with obesity in adolescents. Sleep quality can be measured objectively (i.e., sleep latency, sleep, wake after sleep onset, and the number of awakenings greater than 5 min) [16] and subjectively (i.e., a feeling of sleepiness or fatigue upon awakening and throughout the day) [17]. Meta-analytic data suggests an association between poor sleep quality and overweight and obesity among youth [7]. This association persisted independent of sleep duration in some studies [7]. The US Preventative Services Task Force characterizes inadequate sleep as a key risk factor for obesity [18].

Despite the growing support for the link between inadequate sleep and obesity risk, the sleep duration and quality among children and youth are suboptimal worldwide, particularly on weekdays [19]. The American Academy of Sleep Medicine recommends that children between the ages of 6–12 years achieve between 9 and 12 h of sleep for optimal health and 8 to 10 h in teens aged 13–18 years [20]. A study with a multi-cohort, nationally representative sample of more than 270,000 adolescents in the United States found that more than 50% of adolescents aged 15 and 19 years reported less than 7 h of sleep nightly, which is lower than the recommendations for this age group. Further, less than half of adolescents aged 12–19 reported regularly getting more than 7 h of sleep [21]. A systematic review and meta-analysis summarizing findings from studies published during the COVID-19 pandemic (2020 and 2022) showed reduced sleep quantity and quality among children and adolescents [22]. Moreover, children and adolescents with obesity are more likely to report poor sleep quality [23, 24].

Yoong et al. have reviewed the impact of randomized controlled trials (RCT) of interventions with a sleep component on child BMI, diet, and physical activity [25]. While findings from one of the included studies showed significant improvements in BMI, and one showed significant improvements in the sleep outcomes, the pooled results from the meta-analysis did not yield significant effects on BMI. Additional trials have been conducted since this review, thus, we undertook this systematic review and meta-analysis to evaluate the efficacy of sleep interventions, either focusing on sleep duration or sleep quality, or both, for the treatment of overweight and obesity among children and adolescents aged from 5 to 17 years. Findings from this study will elucidate the relevance of sleep as an intervention for treating childhood obesity.

## Methods

### Protocol registration

The protocol for this review was registered with the international prospective register of systematic reviews (PROSPERO; Registration ID: CRD42021233329). This systematic review followed the Preferred Reporting Items for Systematic Reviews and Meta-Analyses (PRISMA) guidelines. We prespecified change in body mass index (BMI) (including BMI SDS units, and BMI percentile) as the primary outcome. The secondary outcomes of interest included change in body composition using validated anthropometry measurements (not self-reported) such as skinfold thickness, bioelectrical impedance, waist circumference, and dual-energy X-ray absorptiometry (DEXA); reduction in morbidity, changes in reported or quantified (i.e., accelerometers) sleep duration, changes in self- or parent-reported sleep quality, either qualitatively (i.e., questionnaires) or quantitatively (i.e., accelerometer-defined sleep efficiency); changes in self- or parent-reported daytime sleepiness; changes in biochemical measures of circadian phase (e.g., melatonin); cost of intervention; intervention adherence and compliance; change in health-related behavior (diet, physical activity); change in health-related quality of life; change in adverse events; and satisfaction with care outcome.

### Eligibility criteria

Studies were included if they met the following criteria: (1) Study participants youth with overweight or obesity with a mean age between 5 and 17 at the start of the intervention; (2) Study design was a randomized controlled trial or quasi-randomized trial (including individual and cluster randomized) focusing in the treatment of overweight and obesity in children and adolescents which includes a sleep behavior intervention alone or as one component of a multi-component intervention; (3) Intervention comparator (i.e., control group treatment) was no treatment or wait-list control, usual care, or a separate concurrent intervention (e.g., head to head trials); and (4) Article was published in English. There was no restriction placed on the time when studies were published, where the studies were conducted (i.e., country), where the intervention was delivered, who delivered the intervention, or the duration of the intervention.

### Literature search

A search strategy was developed by the research team (HVB, CJV, RF) with support from a library scientist (Supplemental Table 1). The first literature search was run on January 10, 2021 on the following databases: MEDLINE/PubMed, EMBASE/Ovid, PsycINFO/EBSCO, The Cochrane Library [comprising the Cochrane Database of Systematic Reviews, the Cochrane Central Register of Controlled Trials (CENTRAL), and the Cochrane Methodology Register], Web of Science Core Collection/Web of Science, SciELO/Web of Science, and CINAHL/EBSCO. The following sources were also searched for eligible studies: Global Index Medicus, the reference lists of eligible studies, and the conference proceedings of select obesity and sleep societies. On January 10, 2021, the initial search was run in duplicate by two members of the research team (HVB, CJV) and results from one member (HVB) were saved for further screening. The search was re-run on all databases described above on May 12, 2022, for articles published since January 10, 2021, after consulting with an evidence synthesis librarian at the home university of the first author. The references of all included studies were reviewed to identify any potentially missed studies based on the inclusion and exclusion criteria.

### Study selection

Searches were de-duplicated using Zotero and uploaded into Covidence for the title and abstract screening. Title and abstract screening were completed by at least two members of the research team (RF, HVB, CJV, JG, and RL). Review authors were blinded to each other’s decision during screening. Disagreements regarding the inclusion of articles between review authors were resolved by team consensus. The full-text review was subsequently conducted in Covidence in duplicate following the same blinding approach described above. Data from duplicate or companion publications stemming from a single protocol were merged as one single dataset/study for analysis. If consensus for any study was not achieved through discussion and analysis of the manuscripts, a third reviewer was consulted to achieve consensus (LE). All studies included and excluded at each stage of the review are presented in alignment with PRISMA reporting requirements [26].

### Data extraction

Two review authors independently extracted the following categories of data from all included studies using a standardized data extraction template: source, eligibility, methods, study design details, participant information, interventions, outcomes, results, and miscellaneous information such as funding source, key conclusion, and other comments. Study authors were contacted when necessary to request information that was not provided in the acquired published articles or publicly available databases.

### Risk of bias

**Fig. 1:**
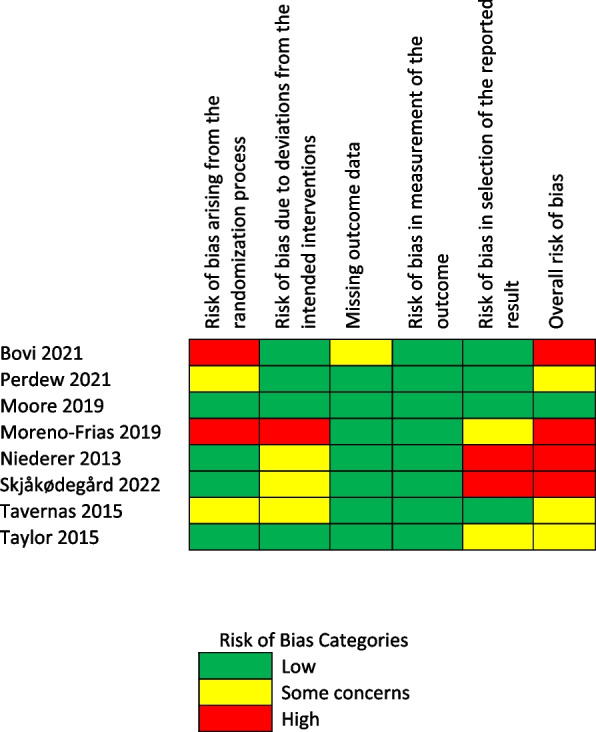
Methodological quality of included studies (Risk of bias)

For all included studies, a risk of bias assessment was conducted by two research team members using the Cochrane Risk of Bias assessment tool (RoB2) [27]. The RoB2 covers five evaluation domains: selection bias, performance bias, detection bias, attrition bias, and reporting bias. Each domain and overall risk of bias can be judged as “low risk of bias”, “some concerns”, or “high risk of bias”. Disagreements in evaluating the risk of bias were resolved by team consensus. When a consensus was not reached, the disagreements were resolved by a third reviewer (LE). Data on the results of the risk of bias assessment are shown in Fig. 1.

### Meta-analytic strategy

Stata 17 was used to conduct a quantitative synthesis of the studies that met inclusion criteria. A meta-analysis was conducted using a random-effects model to estimate the effect of interventions with a sleep component on body composition outcomes (i.e., BMI). A Chi-squared test was used to examine heterogeneity and I^2^ statistic [28] was observed to assess if variation across studies was due to heterogeneity.

## Results

### Search results and selection of studies

**Fig. 2:**
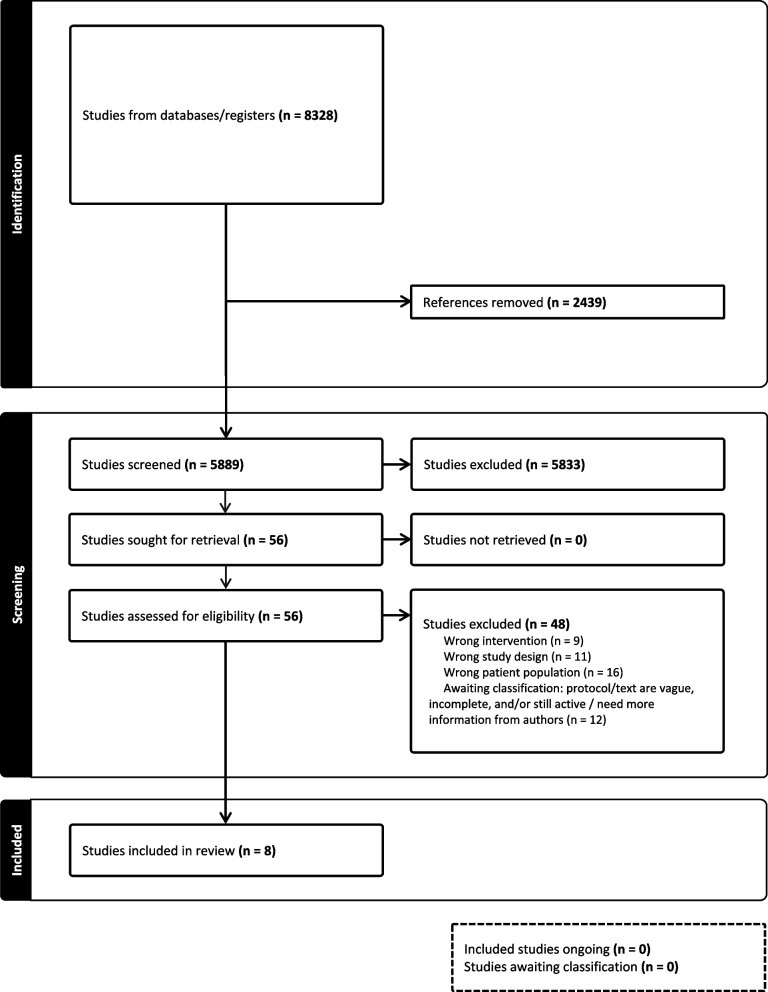
PRISMA Flowchart

After removing 2439 duplicates, a total of 8328 articles were imported for screening. Title and abstract screening resulted in 56 articles for full-text review, of which 8 studies were included. A flowchart detailing the screening process is in Fig. 2.

### Study characteristics

The eight studies included 2231 participants and were conducted in 7 countries, including the United States, Switzerland, New Zealand, Mexico, Canada, Italy, and Norway. Two studies had a quasi-experimental design, one of which was intended as an RCT but had to adopt a nonrandomized protocol during the course of the study due to low recruitment and logistical challenges [29]. The other 7 studies followed the RCT design (five were randomized on the individual level and two were randomized on the cluster level). One RCT excluded enrolled participants based on a weekly assessment of adherence to the protocol. Participants who failed to complete at least 80% of the prescribed diet and sleep intervention (*N* = 56, 51.2%) were excluded and replaced by new participants [30]. All but one study were multi-component lifestyle interventions with a sleep component, ranging from 4 weeks to 3 years in length. One study had sleep as a stand-alone intervention [30]. Besides sleep, factors accounted for in the interventions included diet, physical activity, sedentary activity, media use, stress management, parenting, and screen time. All but one study were family-based interventions involving at least one parent in the education component of the interventions. A description of the intervention characteristics can be found in Table 1.

**Table 1:** Results of individual studies included in the current review

Author name	Intervention description	Variable manipulated	Did sleep improve?	Intervention duration	Study Design	N randomized	Sample	Follow-up duration	Outcome(s) assessed	Findings
***Sleep protocol administered as a stand-alone intervention***										
Moreno-Frias (2019) [29]	Participants were instructed to restrict 500 kcal from their usual diet. Participants in the intervention group were instructed to extent sleep duration of 1 h with incremental daily increases in addition to the calorie restriction. The intervention group also received recommendations on sleep hygiene.	Sleep and diet	Yes. Sleep time (*p* < .02), sleep time at weekend (*p* < .035), sleep efficiency (*p* < .03) improved in the control group; sleep time (*p* < .000001), sleep time at midweek days (*p* < .00004) and at weekend (*p* < .00004), time in bed (*p* < .00001), time awake in bed (*p* < .011), and sleep efficiency (*p* < .006) improved in the intervention group.	4 weeks	RCT	108	Adolescents (*n* = 52) aged 14–18 years with a BMI > 30 kg/m2 corresponding to adult values.	4 weeks	Weight, waist circumference, energy consumption, sleep time, sleep time at midweek day, sleep time at weekend, time in bed, time awake in bed, sleep efficiency, glucose, HDL, Non-HDL cholesterol, triglycerides, leptin, insulin, HOMA-IR, 6-Sulfatoxymelatonin, cortisol, IL-6, and TNF-a	In the control group, energy consumption and weight decreased; sleep time and sleep efficiency increased. In the intervention group, energy consumption, weight, waist circumference, IL-6, HOMA-IR, and insulin level decreased; sleep duration and sleep efficiency increased. The decrease in weight and waist circumference was greater in the intervention group compared to the control group.
***Multicomponent interventions***										
Moore (2019) [32]	Children and one of their parents were randomized into one of the three groups: (1) Healthy Change intervention, (2) System Change intervention, and (3) education-only control group for three years. The intervention groups, despite being based on different theories, both received small group discussions and individualized coaching. Additional coaching was provided to children at the highest risk for excessive body weight or weight gain.	Diet, physical activity, sedentary activity, sleep, and stress management	No (only measured change)	3 years	RCT	360	Children (*n* = 360, entering 6th grade at baseline) with BMI > = 85th percentile and one of their parents or guardians. Participants were primarily African American (77%) and had a family income of < 25,000 per year.	3 years	BMI, waist circumference, tricep skinfold thickness, dietary intake, physical activity, sleep, fitness, blood pressure, and a set of cardiometabolic variables	No differences were found in any outcome variables between the three study groups.
Niederer (2013) [33]	Children participated in weekly physical activity sessions and lessons on nutrition, media use, and sleep. Parents participated in discussions on physical activity, nutrition, and media use. Teachers received training on the intervention content. The school environment was modified to promote physical activity. The control group continued their regular school physical activity curriculum.	Physical activity, nutrition, media use, and sleep	Not measured	9.5 months	Cluster RCT	655	Children from preschool classrooms with a > 40% prevalence of (predominantly German and French) migrants in Switzerland	9.5 months	BMI, aerobic fitness, sum of four skinfolds (SF), waist circumference	The intervention had a significant effect on SF and motor agility for both children with overweight and normal weight. Aerobic fitness only improved among children with normal weight. Children with overweight benefited more from the intervention on waist circumference compared to those with normal weight. Children with low fit benefited more on BMI, SF, and waist circumference from the intervention compared to children with normal fit.
Taylor (2015) [35]	Children and parents participated in a tailored program that consisted of consulting sessions with a multidisciplinary team for a total of 6–7 h over 2 years. The families in the control group received 45–75 min of consulting with a researcher over the 2-year study period.	Parenting, dietary intake, and physical activity	No	2 years	RCT	271	Children aged 4–8 years with BMI > = 85th percentile and their parents	2 years	BMI (and z-score), waist circumference, waist to height ratio, percentage fat, parental feeding practices, child behavior, dietary intake, home food availability, physical activity, sleep	Children in the intervention group had a lower BMI, BMI z-score, waist circumference, and waist to height ratio, and were more physically active than those in the control group. Parents in the intervention group reported higher fruit and vegetable intake, lower noncore food intake, less noncore food present in the home than those in the control group.
Perdew (2021) [30]	Children and parents participated in a community-based program that consisted of 10 weekly 90-minute group educational sessions, 4 community-based activities and the interactive web-portal. The control group had 4 group educational sessions and full access to the web-portal.	Physical activity, diet, sleep hygiene, and parenting	Not measured	10 weeks	Quasi-experimental (RCT failed due to low recruitment)	71	Children aged 8–12 years at or above 85th percentile and their parents	10 weeks	BMI z-score, moderate-to-vigorous physical activity, screen time, sedentary behavior, child dietary behaviors, parental support for healthy eating and physical activity, and self-regulation for healthy eating and physical activity support.	Children in the intervention group showed improved moderate-to-vigorous physical activity level, parental support for healthy eating and physical activity, self-regulation for healthy eating behaviors and physical activity. No differences were observed in BMI z-score between the two groups.
Taveras (2015) [34]	Families (via clinicians or clinicians and health coaches) were provided educational materials about screen time, sugar-sweetened beverages, physical activity, and sleep. Another intervention group received additional individualized health coaching. The control group received usual care according to their pediatric office.	Screen time, sugar-sweetened beverages, physical activity, and sleep	Not measured	1 year	Cluster RCT	549	Children aged 6-12.9 years with a BMI > = 95th percentile and their parents	1 year	BMI (and z-score) and quality of care	Children in the two intervention groups had a smaller mean increase in BMI and BMI z-score compared to those in the control group. The improvements in BMI were greater in the intervention group without the health coaching.
Delli Bovi (2021) [34]	Families participated in a personalized mobile messaging intervention with (IG2) and without (IG1) additional monthly in-presence recall visits. The messages focused on healthy behavior and encouragement to reinforce the behavior. The control group received usual care.	Sugary drinks, fruit and vegetables consumption, breakfast, meal portions, screen-time, physical activity, and sleep	No (hr)	24 weeks	Quasi-experimental	103	Children aged 6–14 years with a BMI > 95th percentile and their parents	24 weeks	BMI (and z-score), waist circumference, neck circumference, blood pressure, obesity-related acanthosis nigricans (AN), sleep duration, physical activity, sedentary behavior, and diet	Children in IG1 had greater improvements in BMI, BMI z-score, and reduction of waist and neck circumference excess compared to the control group at 3 months; no differences at 6 months. Children in IG2 had greater improvements in BMI, BMI z-score, blood pressure, and degree of AN compared the control group at 3 months; the improvements in BMI and AN persisted at 6 months. Compared to the IG1 group, IG2 group showed greater improvements in BMI z-score, waist circumference, and degree of AN.
Skjåkødegård (2022) [36]	Families participated in a family-based behavioral social facilitation treatment (FBSFT) delivered at an obesity outpatient clinic which included 17 individual family sessions. The control group received a personalized plan for healthy behavior change and was encouraged to participate in monthly counseling sessions with nurses.	Diet, sleep, physical activity, sedentary behavior, and sleep	Yes. There was a significant difference in changes in sleep timing (mid-sleep time) from pre- to post-treatment (-26.3 min, *p* = .037) between the intervention and control groups.	178 ± 47 days	RCT	114	Children aged 6–18 years with BMI > = 35 kg/m2 or BMI > = 30 kg/m2 in the presence of weight-related comorbidities	18 months	BMI (and its standard deviation score), sleep, physical activity, and diet	There were significant differences in changes in BMI SDS (*p* < .001), and %IOTF-25 (*p* < .001) from pre- to post-treatment between the intervention and control groups. BMI SDS (*p* < .001) and %IOTF-25 (*p* < .001) decreased in the intervention group from pre- to post-treatment.

### Quality assessment

The overall risk of bias was rated as “low” for one study [31], “some concerns” for three studies [29, 32, 33], and “high” for four studies [30, 34–36]. The risk of bias raised from the randomization process is related to the quasi-experimental design [29, 34], using a pseudo-random number generator to assign participants [32], and the substitution of nonadherent participants with new participants [30]. Deviations from the intended interventions were observed, largely due to a lack of blinding participants and intervention-delivering staff from the intervention groups [32, 35, 36]. Some concerns were observed for attrition bias due to substantial loss-to-follow-up without sufficient explanations for drop-out reasons [34]. Reporting bias exists in four studies, related to deviations from the intended analysis protocol [33, 35, 36] and a lack of preregistration and protocol [30].

### Intervention effect

#### Body weight and composition

All but one study [30] reported at least one of the BMI measures (BMI, BMI z-score, BMI standard deviation scores). Waist circumference was measured in five studies. Other anthropometric measures included tricep skinfold thickness, the sum of four skinfolds, waist-to-height ratio, percentage body fat, and neck circumference. Only one study [30] studied sleep in isolation compared to a multicomponent format. This study, by Moreno-Frias et al. [30], showed a significantly greater weight (*p* < .04) and waist circumference (*p* < .0009) reduction in the experimental group (*n* = 25) vs. control (*n* = 27) after 4 weeks of intervention. Other studies included sleep as part of a multicomponent intervention but did not isolate the sleep component against a similar control group without the sleep component. In the Ballabeina study [35], at 9.5 months post-intervention children who were overweight (regression coefficients − 2.19, 95%CI [-3.18, -1.20], *p* < .0001) showed a greater reduction in waist circumference compared to children (-0.67 [-1.24, -0.11], *p* = .02) who were normal weight (intervention-group x BMI-group, *p* = .001). Improvements in the sum of four skinfolds were observed in both the overweight (-3.63 [-6.45, 0.81], *p* = .01) and normal groups (-2.46 [-3.91, -1.01], *p* = .0001). No change in BMI was observed in either group. Taylor et al. [33] found significantly greater improvements in BMI (difference − 0.34, 95%CI [-0.65, -0.03]), BMI z-score (-0.12 [-0.20, -0.04]), waist circumference (-1.5 cm [-2.5, -0.5]), and waist-to-height ratio (-0.01 [-0.02, -0.00]) in the experimental group (*n* = 96) compared to the control group (*n* = 97) at 24 months. Taveras et al. [32] found increases in BMI and decreases in BMI z score units in all groups (usual care, clinical decision support (CDS), CDS plus health coaching) at 12 months. The increase in BMI was the greatest in the usual care group (+ 1.2) (*n* = 171), followed by the CDS plus health coaching group (+ 0.9) (*n* = 164), and the CDS group (+ 0.7) (*n* = 183). The two experimental groups had a greater reduction in BMI z-score units than the usual care group, though the greatest reduction was observed in the group without health coaching (-0.06, 95%CI [-0.11, − 0.02]). Bovi et al. [34] compared three groups in two phases, standard treatment (CG1.1 and CG1.2), control plus personalized messaging (IG1.1), and IG1.1 plus monthly recall visits (IG1.2). IG1.1 vs. CG1.1 and IG1.2 vs. CG1.2 were compared in phases 1 and 2, respectively. At 3 months, greater improvements in BMI, excess waist circumference, and excess neck circumference were observed in IG1.1 (*n* = 24) compared to CG1.1 (*n* = 25). However, the improvements did not sustain at 6 months. BMI mean change at 6 months was significantly different between IG1.2 (-4.6, *n* = 30) and CG1.2 (+ 2.7, *n* = 24) (*p* = .003). Skjakodegard et al. [36] found significant differences in mean change in BMI standard deviation scores (0.19 units, *p* < .001) and the proportion of participants above the International Obesity Task Force cut-off for overweight (5.48%, *p* < .001) between the experimental (*n* = 59) and control (*n* = 55) groups at 12 months. No statistically significant differences were found in BMI measures and anthropometric measures posttreatment in the other studies [29, 31].

#### Sleep outcomes

Out of the five studies that assessed sleep, two found improvements in sleep. Moreno-Frias et al. collected self-report sleep-related outcomes by telephone interviews, including sleep duration, time in bed, time awake in bed, and sleep efficiency (percent of sleep time concerning the total time in bed). Significant improvements in overall sleep duration, weekend sleep duration, and sleep efficiency were observed in both the experimental (*n* = 25) and control (*n* = 27) groups at 4 weeks. The experimental group also showed significant improvements in weekday sleep duration, time in bed, and time awake in bed. However, participants who did not adhere to the sleep extension intervention by at least 80% were swapped with new participants during the study [30]. Skjakodegard et al. [36] measured sleep duration and timing using wrist-worn accelerometers (Actiwatch 2) for 7 days. Sleep timing was defined as the midpoint between sleep onset time and wake-up time. There was a significant difference in mean changes in sleep timing from baseline to posttreatment between the experimental (*n* = 59) and control (*n* = 55) groups (− 26.3 min, *p* = .037) at 12 months. In the study targeting urban youth [31], sleep durations on weekends and weekdays were measured using accelerometers (GT3X + monitor) hip-worn by children for 7 days. Parents reported on children’s sleep quality and duration. No significant differences were found between the experimental and control groups in these outcomes. Taylor et al. [33] measured sleep duration using hip-worn accelerometers (ActiGraph GT3X) for 7 days and 8 nights. No evidence of a difference in sleep duration was found when comparing the two groups after the intervention (*p* = .317)^35^. In the PediaFit study [34], participants self-reported the number of hours of sleep per night. There were no significant differences between the experimental and the control groups at three months (*p* = .55) or six months (*p* = .8).

#### Dietary outcomes

Five studies reported dietary outcomes. Intakes of fruits, vegetables, and sugary beverages were the most reported variables. Children’s Dietary Questionnaire [37] was completed by parents to estimate child intakes of “recommended” foods (fruits, vegetables, water, and reduced fat products), and “discouraged” foods (high fat/sugar foods/noncore foods, and sweetened beverages) over the past week in Taylor et al. [33]. Results showed that children in the experimental group (*n* = 89) had greater improvements in fruit and vegetable intake (difference 1.0, 95% CI [0.0, 2.1]) and noncore food intake (-0.3, [-0.5, -0.0]) compared to those in the control group (*n* = 92) at 24 months. However, the 95% confidence intervals for both outcomes contain zero, indicating that the evidence for an effect is weak. At 3 months, Bovi et al. [34] found significant differences between IG1.1 (*n* = 12) and CG1.1 (*n* = 6), as well as between IG1.2 (*n* = 12) and CG1.2 (*n* = 6) in sugary drink consumption (IG1.1 vs. CG1.1 *p* = .002, IG1.2 vs. CG1.2 *p* = .02) and fruit and vegetable consumption (IG1.1 vs. CG1.1 *p* = .040, IG1.2 vs. CG1.2 *p* = .04). However, there was no difference in these dietary outcomes at 6 months except for fruit and vegetable consumption between IG1.2 (*n* = 9) and CG1.2 (*n* = 2) (*p* = .02). No significant intervention effect on dietary outcomes was found in other studies.

#### Physical activity

Three studies measured physical activity using accelerometry data, and two studies used self-report survey data. The accelerometry data collected by Taylor et al. [33] showed that children in the experimental group (*n* = 89) were more physically active (higher mean counts per minute) than those in the control group (*n* = 92) (difference 60, 95% CI [4, 115]) at 24 months. No significant differences were found in the moderate-to-vigorous-physical-activity (MVPA) level. Perdew et al. measured MVPA using the Physical Activity Questionnaire for Older Children (PAQ-C). Children in the experimental group (*n* = 48) significantly increased their weekly MVPA duration (0.75 ± 1.5 min) while the opposite is true for those in the control group (*n* = 23) (-0.74 ± 1.6 min) (*p* = .001) at 10 weeks [29]. Parents in the study led by Bovi et al. [34] reported minutes of child physical activity per day per week. The findings showed a significant difference in change in weekly physical activity duration between IG1.2 (*n* = 12) participants (71.85 ± 118.0 min) and CG1.2 (*n* = 6) participants (-30 ± 111.0 min) at 3 months (*p* = .03). There was no between-group difference at 6 months. No significant intervention effect on physical activity was found in other studies.

#### Meta-analysis

**Fig. 3:**
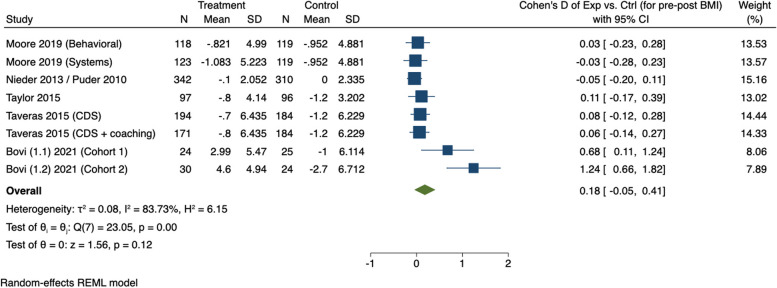
Forest plot depicting the effect of obesity-related interventions with a sleep component on body mass index

Table 2 summarizes the modeling results from the random-effects meta-analysis, with the corresponding forest plot shown in Fig. 3. A total of 2 studies were excluded from the meta-analysis because these studies did not adopt the same measure for an outcome (i.e., the primary outcome measure was not comparable to BMI) [29, 30]. Five out of the eight studies that met inclusion criteria were included in the meta-analysis, which compared effect sizes from behavioral or multi-component interventions with a sleep component relative to a control group providing standard of care [31–35]. Compared with the control group, there was no significant overall effect on children’s BMI as a result of participation in an intervention with a sleep component (Cohen’s d = 0.18, 95% CI= -0.04, 0.40, Z = 1.56, *P* = .11). Lastly, there was substantial heterogeneity observed across studies included in the meta-analysis (Tau^2^ = 0.08; X^2^ = 23.05, df = 7; I^2^ = 83.73%).

**Table 2:** Results from meta-analyses using a random effects model

Study	Effect size	[95% conf. interval]	% Weight
Moore 2019 (Behavioral) [32]	0.026	-0.227, 0.280	13.53
Moore 2019 (Systems) [32]	-0.026	-0.277, 0.225	13.57
Nieder 2013 / Puder 2010 [33]	-0.046	-0.199, 0.108	15.16
Taylor 2015 [35]	0.108	-0.174, 0.389	13.02
Taveras 2015 (CDS) [34]	0.079	-0.123, 0.280	14.44
Taveras 2015 (CDS + coaching) [34]	0.063	-0.145, 0.271	14.33
Bovi (1.1) 2021 (Cohort 1)	0.676	0.109, 1.243	8.06
Bovi (1.2) 2021b (Cohort 2)	1.242	0.664, 1.821	7.89
**Test of heterogeneity**: Tau^2^ = 0.08; Chi^2^ = 23.05, df = 7; I^2^ = 83.73%			
**Test for overall effect**: Z = 1.56 (*P* = .11)

## Discussion

Overall, our systematic review included eight studies across seven countries. There were mixed results on the effect of sleep interventions across included studies on BMI, other weight-related outcomes, diet, physical activity, and sleep, and weight status. The result of the meta-analysis did not identify a significant effect of sleep interventions on the primary study outcomes. Except for one study at low risk of bias, others were rated as ‘some concerns’ or high risk of bias. Findings from this study highlight the need for additional RCTs isolating sleep as a component, focusing on children and adolescents living with overweight and obesity.

A previous systematic review [25] found no significant impact of sleep interventions on BMI among children and adolescents with normal weight under the age of 18. The inconsistencies in obesity metrics used present challenges in interpreting the overall intervention impact. One study found a reduction in adiposity measures but not BMI [35]. There is no evidence supporting the intervention effect on child dietary behaviors in this review. Only one included study demonstrated significant improvements in dietary intake (i.e. fruit and vegetable intake) following the intervention [34]. Yoong et al. observed some positive intervention effects on child diet, but were unable to examine the intervention impact on physical activity because only one included study collected physical activity data. In the current review, three studies showed improvements in physical activity after the intervention. However, three different instruments, including subjective and objective measures, were used in these studies. Hence, more research using consistent physical activity measures is needed to explore the relationship between sleep interventions and physical activity further.

The current evidence base has several limitations. First, the integration of sleep into interventions is not consistent across the included studies. Only one study had sleep as a stand-alone intervention, other interventions included sleep as part of the education material along with other variables such as diet, physical activity, and parenting. Improvements in sleep were observed in only one study that included sleep as an education component as a part of the intervention. This might suggest that education alone is not sufficient to initiate and maintain sleep behavior change. As such, more studies that include sleep as a stand-alone intervention are needed, which will then provide insight into the relationship between sleep and obesity treatment. Second, sleep was assessed differently among included studies. Three studies did not assess any sleep outcome variables. Without this data, it is challenging to delineate the relationship between sleep interventions and changes in obesity metrics. Third, of studies (*n* = 3) that reported sleep duration at baseline, on average participants were meeting national requirements for sleep, ranging from 7.6 h [36] to 9.5 h [33]. Given that, it is plausible that the lack of intervention effect was related to a ceiling effect in sleep improvement and subsequently change in obesity metrics. For this reason, future interventions should consider enrolling youth with obesity and sleep insufficiency/poor sleep at baseline. Further, only two studies [33, 36] used objective instruments (i.e. accelerometry) to measure sleep. In some studies that relied on self-report sleep outcomes, it was not clear whether the survey or diary was completed by the parent or the child. Reporting bias, particularly overestimation, in self-report sleep duration has been documented in adults. The correlation between self-report duration and accelerometry data is moderate [38] to weak [39]. Although the agreement between subjective and objective measures of sleep duration in children is unknown, it is reasonable to suspect a similar extent of inconsistencies as observed in adults. Whether it is the parent or child reporting sleep duration could also introduce variability in the data collected. Given that, future studies should incorporate objective measures of sleep duration and report who reported the data if using subjective instruments. Lastly, only two studies measured sleep quality by sleep efficiency [30] and sleep timing [36]. Both sleep duration and quality are linked to sleep adequacy and obesity in youth [7], therefore, measuring sleep quality in addition to duration is needed in future studies to determine the efficacy of lifestyle interventions on improving sleep as a treatment for children and adolescents living with overweight and obesity.

This study has strengths and limitations. The study is strengthened by our emphasis on sleep interventions, rather than lifestyle interventions, which reveals the paucity of interventions that isolate sleep and underscores a need for future sleep-focused interventions. Lastly, to our knowledge, this is one of the first reviews focusing on interventions to treat overweight and obesity in childhood populations. We also acknowledge the high heterogeneity in our meta-analysis from the studies that met inclusion criteria in our study. Because of the variety of designs used, we are not confident that a meta-analysis (or subgroup analyses) is appropriate to summarize the literature on this research question at this time. We however present the results of the overall meta-analysis for readers to make appropriate inferences based on our results. Another limitation worth acknowledging are the logistical challenges throughout stages of this review involving scientists from various disciplines and across at least 3 continents. Finalizing this review took much collaboration and documentation to ensure the quality of the process remained optimal. Evidence from this systematic review shows it remains unclear whether sleep is an effective component of a lifestyle intervention or as a stand-alone intervention on overweight or obesity for children aged 5–17 years.

## Conclusions

To conclude, this study finds no evidence of a significant effect of sleep interventions on BMI other weight-related outcomes across included studies for children aged 5–17 years with overweight or obesity. Future intervention studies with rigorous RCT design that incorporate objective measures of sleep are needed to inform guideline recommendations on sleep for youth with overweight and obesity.

### Supplementary Information

**Additional file 1.**

## Data Availability

All data generated or analyzed during this study are included in this published article.
