# Supplementary material for: The efficacy of sleep lifestyle interventions for the management of overweight or obesity in children: a systematic review and meta-analysis
Source: BMC Public Health. 2024 Jan 29;24:321. doi: 10.1186/s12889-024-17708-6 (PMC10825984; doi:10.1186/s12889-024-17708-6)
Supplement: Supplementary file 1 — Additional file 1. [file 12889_2024_17708_MOESM1_ESM.docx]

Search Strategy Update Documentation

**Search summary**

| **Database Name** | **Platform** | **Date of Search** | **# of results** |
| --- | --- | --- | --- |
| 1. MEDLINE | Pubmed | 5/12/2022 | 281 |
| 2. EMBASE | Ovid | 5/11/2022 | 152 |
| 3. PsycINFO | EBSCO | 5/12/2022 | 27 |
| 4. CINAHL | EBSCO | 5/12/2022 | 189 |
| 5. Cochrane Library | n/a | 5/12/2022 | 161 |
| 6. Web of Science Core Collection | Web of Science | 5/12/2022 | 133 |
| 7. Scielo | Web of Science | 5/12/2022 | 25 |
| 9. Global Index Medicus | n/a | 5/12/2022 | 171 |

**Total Records = 1139**

**Total Records after deduplication =**

# [Update- May 12, 2002]

# Primary search strategy – **DO NOT EDIT**, CONSIDERED FINAL

**1. MEDLINE / Pubmed**

Date of Search: 5/12//2022

Number of results: 281

Original google doc of search strategy [here](https://docs.google.com/document/d/1ogkMjCP1pecZbRQCS29gmVBbo6ZSFaa88sE6qIxbv1A/edit?usp=sharing).

| **Concept** | **#** | **Search string** | **# of results** |
| --- | --- | --- | --- |
| **Population MeSH term (age)** | **1** | Child[MeSH] OR adolescent[Mesh] | 3,264,812 |
| **Population keyword (age)** | **2** | Child*[tiab] OR adolescen*[tiab] OR teen*[tiab] OR young adult*[tiab] OR youth[tiab] OR pediatric*[tiab] OR paediatric*[tiab] OR school age*[tiab] OR kid[tiab] OR boy[tiab] OR girl[tiab] OR juvenile[tiab] OR teen[tiab] OR teenager*[tiab] OR schoolchild*[tiab] OR preteen*[tiab] OR youth*[tiab] OR young person[tiab] OR young people[tiab] | 2,198,925 |
|  | **3** | #1 OR #2 | 3,264,812 |
| **Population/obesity Mesh** | **4** | Overweight[mesh] | 254,641 |
| **Population/obesity tiab** | **5** | Overweight[tiab] OR over weight[tiab] OR over-weight[tiab] OR obes*[tiab] OR adipos*[tiab] | 451,466 |
|  | **6** | #4 OR #5 | 497,210 |
| **Intervention Mesh** | **7** | Sleep[MESH] OR Circadian clocks[MESH] OR circadian rhythm[MESH] | 158,676 |
| **Intervention Tiab** | **8** | Sleep*[tiab] OR wake*[tiab] OR evening[tiab] OR eveningness[tiab] OR drows*[tiab] OR bed[tiab] OR bedtime[tiab] OR bedroom[tiab] OR polysomnography[tiab] OR circadian[tiab] OR melatonin[tiab] OR  awake[tiab] OR fatigue[tiab] OR bedtime[tiab] OR morning[tiab] OR morningness[tiab] OR circadian[tiab] OR polysomnography[tiab] OR insomnia*[tiab] OR actigraph*[tiab] | 568,587 |
|  | **9** | #7 OR #8 | 609,431 |
| **RCT filter** | **10** | randomized controlled trial [pt] OR controlled clinical trial [pt] OR randomized [tiab] OR placebo [tiab] OR drug therapy [sh] OR randomly [tiab] OR trial [tiab] OR groups [tiab] | 5,423,603 |
|  | **11** | #3 AND #6 AND #9 AND #10 | 1,900 |
| Date filter for updated search | **12** | ("2021/01/10"[CRDT] : "3000"[CRDT] OR "2021/01/10"[EDAT] : "3000"[EDAT] OR "2021/01/10"[MHDA] : "3000"[MHDA]) | 2,881,307 |
|  | **13** | #11 AND #12 | 281 |

# Convert the search strategy syntax for each other database searched

**2. EMBASE/Ovid <1974 to 2022 May 11>**

Date of Search: 05/11/2022

Number of results: 152

| **#** | **Search string** | **# of results** |
| --- | --- | --- |
| 1 | exp child/ or exp adolescent/ | 3695144 |
| 2 | (child* or adolescen* or teen* or "young adult*" or youth or pediatric* or paediatric* or "school age*" or kid or boy or girl or juvenile or teen or teenager* or schoolchild* or preteen* or youth* or "young person" or "young people").mp. | 4336768 |
| 3 | 1 OR 2 | 4821061 |
| 4 | exp obesity/ | 592899 |
| 5 | (overweight or over weight or over-weight or obes* or adipos*).mp. | 784786 |
| 6 | 4 OR 5 | 827572 |
| 7 | exp sleep/ or exp circadian rhythm/ | 341980 |
| 8 | (sleep* or wake* or evening or eveningness or drows*: or bed or bedtime or bedroom or polysomnography or circadian or melatonin or awake or fatigue or morning or morningness or circadian or polysomnography or insomnia* or actigraph).mp. | 1122429 |
| 9 | 7 OR 8 | 1147148 |
| 10 | (random$ or placebo$ or single blind$ or double blind$ or triple blind$).ti,ab. | 1925523 |
| 11 | RETRACTED ARTICLE/ | 11099 |
| 12 | 10 or 11 | 1936148 |
| 13 | (animal$ not human$).sh,hw | 4639945 |
| 14 | (book or conference paper or editorial or letter or review).pt. not exp randomized controlled trial/ | 5566059 |
| 15 | (random sampl$ or random digit$ or random effect$ or random survey or random regression).ti,ab. not exp randomized controlled trial/ | 141150 |
| 16 | 12 not (13 or 14 or 15) | 1475394 |
| 17 | 3 and 6 and 9 and 16 | 989 |
| 18 | limit 17 to dc=20210110-20220511 | 152 |

**3. PyscINFO/EBSCO**

Date of Search: 5/12/2022

Number of results: 27

Citation Alert Account Info:

| **#** | **Search string** | **# of results** |
| --- | --- | --- |
| S1 | TX(child* OR adolescen* OR teen* OR "young adult*" OR youth OR pediatric* OR paediatric* OR "school age*" OR kid OR boy OR girl OR juvenile OR teen OR teenager* OR schoolchild* OR preteen* OR youth* OR "young person" OR "young people") | 1,839,929 |
| S2 | DE(overweight) | 7,271 |
| S3 | TX (“Overweight” OR “over weight*” OR “over-weight” OR obes* OR adipos*) | 56,640 |
| S4 | S2 OR S3 | 56,640 |
| S5 | DE(sleep OR human biological rhythms) | 37,995 |
| S6 | TX(Sleep* OR wake* OR “evening” OR “eveningness*” OR drows* OR bed* OR bedtim* OR bedroom* OR “polysomnography*” OR “circadian*“ OR melatonin* OR  awake* OR fatigue* OR bedtime* OR morning* OR morningness* OR insomnia* OR actigraph*) | 208,817 |
| S7 | S5 OR S6 | 209,464 |
| S8 | SU.EXACT("Treatment Effectiveness Evaluation") OR SU.EXACT.EXPLODE("Treatment Outcomes") OR SU.EXACT("Placebo") OR SU.EXACT("Followup Studies") OR placebo* OR random* OR "comparative stud*" OR clinical NEAR/3 trial* OR research NEAR/3 design OR evaluat* NEAR/3 stud* OR prospectiv* NEAR/3 stud* OR (singl* OR doubl* OR trebl* OR tripl*) NEAR/3 (blind* OR mask*) | 273,765 |
| S9 | S1 AND S4 AND S7 AND S8 | 439 |
| S10 | Limit: Publication date: 20210101-20220531 | 27 |

<http://resolver.library.cornell.edu/misc/2351079>

**4. CINAHL/EBSCO**

Date of Search: 5/12/2022

Number of results: 189

Citation Alert Account Info:

| **#** | **Search string** | **# of results** |
| --- | --- | --- |
| S1 | MH(“Child+” OR “adolescent+”) | 724,894 |
| S2 | TX(Child* OR adolescen* OR teen* OR young adult* OR youth OR pediatric* OR paediatric* OR school age* OR kid OR boy OR girl OR juvenile OR teen* OR “teenager*” OR schoolchild* OR preteen* OR youth* OR “young person” OR “young people”) | 1,672,817 |
| S3 | S1 OR S2 | 1,675,165 |
| S4 | MH(“Obesity+”) | 110,348 |
| S5 | TX (“Overweight” OR “over weight*” OR “over-weight” OR obes* OR adipos*) | 186,894 |
| S6 | S4 OR S5 | 187,454 |
| S7 | MH (“Sleep+” OR “Chronobiology Disorders+” OR “circadian rhythm+”) | 36,694 |
| S8 | TX(Sleep* OR wake* OR “evening” OR “eveningness*” OR drows* OR bed* OR bedtim* OR bedroom* OR “polysomnography*” OR “circadian*“ OR melatonin* OR  awake* OR fatigue* OR bedtime* OR morning* OR morningness* OR insomnia* OR actigraph*) | 281,549 |
| S9 | S7 OR S8 | 282,695 |
| S10 | TX allocat* random* OR (MH "Quantitative Studies") OR (MH "Placebos") OR TX placebo* OR TX random* allocat* OR (MH "Random Assignment") OR TX randomi* control* trial* OR TX ( (singl* n1 blind*) OR (singl* n1 mask*) ) OR TX ( (doubl* n1 blind*) OR (doubl* n1 mask*) ) OR TX ( (tripl* n1 blind*) OR (tripl* n1 mask*) ) OR TX ( (trebl* n1 blind*) OR (trebl* n1 mask*) ) OR TX clinic* n1 trial* OR PT Clinical trial OR (MH "Clinical Trials+") | 1,637,287 |
| S11 | S3 AND S6 AND S9 AND S10 | 1,092 |
| S12 | ZD (2021* OR 2022* OR "in process") | 1,206,775 |
| S13 | S11 AND S12 | 189 |

<https://www.sign.ac.uk/what-we-do/methodology/search-filters/>

<https://guides.library.cornell.edu/evidence-synthesis/search-strategy>

**5. Cochrane Library**

Date of Search: 5/12/2022

Number of results: 1073 (Trials only) +4 (Cochrane reviews)

Citation Alert Account Info:

| **#** | **Search string** | **# of results** |
| --- | --- | --- |
| 1 | MeSH descriptor: [Child] explode all trees | 61,040 |
| 2 | MeSH descriptor: [Adolescent] explode all trees | 110,030 |
| 3 | (Child* OR adolescen* OR teen* OR “young adult” OR youth OR pediatric* OR paediatric* OR “school age” OR kid OR boy OR girl OR juvenile OR teen OR teenager* OR schoolchild* OR preteen* OR youth* OR “young person” OR “young people”):ti,ab,kw | 327,216 |
| 4 | #1 OR #2 OR #3 | 327,216 |
| 5 | MeSH descriptor: [Overweight] explode all trees | 18,754 |
| 6 | (Overweight OR “over weight” OR “over-weight” OR obes* OR adipos*):ti,ab,kw | 54,869 |
| 7 | #5 OR #6 | 54,948 |
| 8 | MeSH descriptor: [Sleep] explode all trees | 6,371 |
| 9 | MeSH descriptor: [Circadian clocks] explode all trees | 22 |
| 10 | MeSH descriptor: [circadian rhythm] explode all trees | 3,109 |
| 11 | (Sleep* OR wake* OR evening OR eveningness OR drows* OR bed OR bedtime OR bedroom OR polysomnography OR circadian OR melatonin OR  awake OR fatigue OR bedtime OR morning OR morningness OR circadian OR polysomnography OR insomnia* OR actigraph*):ti,ab,kw | 125,606 |
| 12 | #8 OR #9 OR #10 OR #11 | 125,675 |
| 13 | #4 AND #7 AND #12 | 1240 + 4 Cochrane reviews (which were uploaded) |
| 14 | Trials: Date added to CENTRAL trials database filter applied  Reviews: Publication date filter applied. | 161 trials  0 reviews |

**6. Web of Science (Core Collection, Scielo)**

Date of Search: 5/12/2022

Number of results: Core Collection: 133

Scielo 25

Citation Alert Account Info:

| **WoS Core Collection – 5/12/2022 (Exact Search not on)** | | |
| --- | --- | --- |
| **#** | **Search string** | **# of results** |
| 1 | TS=(child* OR adolescen* OR teen* OR young adult* OR “youth” OR pediatric* OR p$diatric* OR school age* OR “kid” OR “boy” OR “girl” OR “juvenile” OR “teen” OR teenager* OR schoolchild* OR preteen* OR youth* OR “young person” OR “young people”) | 3,071,414 |
| 2 | TS=(“overweight” OR “over weight” OR “over-weight” OR obes* OR adipos*) | 609,686 |
| 3 | TS=(sleep* OR wake* OR “evening” OR “eveningness” OR drows* OR “bed” OR “bedtime” OR “bedroom” OR “polysomnography” OR “circadian” OR “melatonin” OR “awake” OR “fatigue” OR “bedtime” OR “morning” OR “morningness” OR “circadian” OR “polysomnography” OR insomnia* OR actigraph*) | 1,086,377 |
| 4 | TS=(randomly OR randomised OR randomized OR "random allocat*" OR RCT OR CCT OR "double blind*" OR "single blind*" OR "double blind*" OR "single blind*" OR trial) | 2,505,999 |
| 5 | #1 AND #2 AND #3 AND #4 | 975 |
| 6 | LD=(2021-01-11/2022-05-12) | 4,822,911 |
| 7 | #5 AND #6 | 133 |

| **Scielo – 1/10/2021** | | |
| --- | --- | --- |
| **#** | **Search string** | **# of results** |
| 1 | TS=(child* OR adolescen* OR teen* OR young adult* OR “youth” OR pediatric* OR p$diatric* OR school age* OR “kid” OR “boy” OR “girl” OR “juvenile” OR “teen” OR teenager* OR schoolchild* OR preteen* OR youth* OR “young person” OR “young people”) | 83,723 |
| 2 | TS=(“overweight” OR “over weight” OR “over-weight” OR obes* OR adipos*) | 13,498 |
| 3 | TS=(sleep* OR wake* OR “evening” OR “eveningness” OR drows* OR “bed” OR “bedtime” OR “bedroom” OR “polysomnography” OR “circadian” OR “melatonin” OR “awake” OR “fatigue” OR “bedtime” OR “morning” OR “morningness” OR “circadian” OR “polysomnography” OR insomnia* OR actigraph*) | 10,952 |
| 4 | #1 AND #2 AND #3 | 154 |
| 5 | LD=(2021-01-10/2022-05-12) | 66,038 |
| 6 | #4 AND #5 | 25 |

**7. Global Index Medicus**

Date of Search: 5/12/2022

Number of results: 171

| **#** | **Search string -** | **# of results** |
| --- | --- | --- |
| 1 | (tw:(child OR adolescent OR teen OR "young adult" OR youth OR pediatric OR paediatric OR boy OR girl OR juvenile OR teenager OR preteen OR youth)) AND (tw:(overweight OR "over weight" OR "over-weight" OR obese OR obesity OR adiposity)) AND (tw:(sleep OR wake OR evening OR eveningness OR drowsy OR drowsiness OR bed OR bedtime OR bedroom OR polysomnography OR circadian OR melatonin OR awake OR fatigue OR tired OR morning OR morningness OR circadian OR insomnia OR actigraph OR actigraphy)) AND ( la:("en")) | 171 |

Note: no date filter was applied as the search string was modified to better fit the syntax of the database.
